# The Influence of Family History of Type 2 Diabetes Mellitus on Positive Health Behavior Changes among African Americans

**DOI:** 10.1155/2020/8016542

**Published:** 2020-02-03

**Authors:** Donny Ard, Naa-Solo Tettey, Shinga Feresu

**Affiliations:** ^1^Department of Surgery, Holy Cross Hospital, Silver Spring, MD 20910, USA; ^2^Department of Public Health, William Paterson University, Wayne 07470, USA; ^3^Department of Public Health, University of Johannesburg, Auckland Park 2006, South Africa

## Abstract

Type 2 diabetes mellitus (T2DM) is a disease that affects the body's ability to metabolize glucose effectively. The disease is predicted to be prevalent in over 300 million people by the year 2030. African Americans (AA) have the highest prevalence rates of type 2 diabetes mellitus (T2DM) in the United States. Lifestyle modification and awareness of risk factors, including family history, are important aspects for prevention of developing T2DM. The purpose of this study was to understand if a family history of T2DM played an influential role in individuals making positive health behavior changes for T2DM prevention. The phenomenological study was grounded in the health belief model and also identified barriers associated with inactivity towards positive health behavior changes. Participants selected for this study were at least 18 years of age, self-identified as AA, self-reported a family history of T2DM, and were not diagnosed with the disease themselves. Transcriptions of twenty face-to-face interviews were analyzed via qualitative research software NVivo Version 12 for Mac. Participants demonstrated a strong awareness of T2DM with an accurate definition of T2DM and explanation of signs, symptoms, and prevention. Participants recognized family history as a risk factor in only 55% of the responses. However, family history played a major role in prevention in the lives of the participants. The participants reflected on personal barriers to health behavior changes and were encouraged to incorporate better life choices in their own lives. This research offers communities, healthcare providers, and stakeholders a better understanding of the importance of family history as a risk factor to T2DM as programs are developed to mitigate health disparities in the AA community.

## 1. Introduction

Diabetes mellitus (DM) is a disease that affects the body's ability to effectively break down sugar for the consumption of energy. There are three types of DM: type 1, type 2, and gestational DM. Type 2 DM (T2DM) deals with the cells of the body not responding to the hormone insulin, the body not producing enough insulin, or both [1]. According to the Centers for Disease Control and Prevention (CDC), diabetes affects approximately 29.1 million individuals each year and is the seventh leading cause of mortality in the United States [2]. The World Health Organization estimates that T2DM will be prevalent among 366 million people by the year 2030, which is a dramatic increase from the 171 million that was reported globally in 2000 (World Health Organization, 2010 as cited in [3]).

African Americans are twice as likely to be predisposed to develop diabetes as their European American counterparts [4]. This disparity has elevated the issue to a national concern. Healthy People 2020, through the goals and objectives for diabetes, recognizes there is a significant health disparity among African Americans diagnosed with T2DM [5]. As it pertains to race and ethnicity, African Americans are 50%-100% more likely to develop T2DM compared to their European American counterparts [6]. Researchers estimate the rate of T2DM to triple by the year 2050 among African Americans [6]. African Americans are more likely to have disproportionate outcomes as they relate to T2DM and are twice as likely to experience diabetes-related blindness, lower limb amputations, and depression [4, 7].

T2DM remains the leading cause of death for African Americans. The overall goal of Healthy People 2020 in this regard is to reduce the number of newly diagnosed individuals with T2DM and contribute to reducing the economic burden it has on individuals and their families [5]. The keys to this reduction in newly diagnosed individuals are within awareness and prevention. Awareness involves understanding the risk factors associated with the disease. The risk factors related to T2DM include obesity, hypertension, heart disease, family history, and ethnicity. Compared with individuals without a family history of T2DM, persons with a family history in any first-degree relative have a 2- to 3-fold increased risk of developing T2DM [8]. The way a person internalizes the importance of family history varies by disease. Individuals becoming aware of their family history and assessing their personal relationship to a disease may result in a positive change in health behavior [9]. McDowell et al. [9] found that individuals who were aware of a family history of prostate cancer were more likely to undergo screening as compared to those who did not have a family history. In another study, Madlensky et al. [10] showed that women who had a strong family history of breast cancer were more likely to take more of a dramatic approach to the disease as it pertains to surgical management. At the same time, they did not report more preventive measures as compared to breast cancer survivors who did not have a family history.

A shift in thinking within the African American community, healthcare providers, and stakeholders on the role of positive health behavior and family history as it pertains to improving the overall outcome of T2DM is needed. How the family history of T2DM is influential as a positive behavior change agent for African Americans was the area of interest for this study. There are several gaps in the literature specifically in looking at the impact that knowledge of family history has on preventive measures in T2DM. This study provides a perspective of African Americans who have a family history of T2DM and how knowledge of this affects lifestyle choices. This research has the potential to shed light on specific barriers that may hinder individuals in making lifestyle changes. This study adds to the discipline of T2DM, African Americans, and community health promotion and education.

## 2. Materials and Methods

### 2.1. Research Design Rationale

To fully understand the influence the knowledge of family history of T2DM has on an individual to engage in positive health behavior changes, a qualitative research method was used. This research method is favored because it allows for interaction with the participants via face-to-face interviews. Focus groups and interviews have been used in prior research that focused on diabetes and some other lived experience. Jagiello and Azulay Chertok [11] illustrated this concept when they looked at women's experiences with early breastfeeding after GDM. Prompting questions were used to initiate conversation in the focus groups and the interviews; detailed accounts from the participants were recorded and analyzed for the research [11].

### 2.2. Theoretical Foundation

The theoretical framework for this research was the health belief model (HBM). The HBM explores the mindsets of individuals and their willingness to make healthy choices. The HBM provides insight on barriers associated with persons who may refuse to involve themselves in any positive changes towards their health [12]. The HBM for this study assisted in examining African Americans with a family history of diabetes and the influence it has had on positive health behavior changes.

### 2.3. Conceptual Framework

The conceptual framework for this research was centered on a phenomenological design. Phenomenology is a broad discipline and method of inquiry in philosophy. German philosophers Edmund Husserl and Martin Heidegger were primarily responsible for its conception. Phenomenology is based on the premise that reality consists of objects and events (“phenomena”) as they are perceived or understood in the human consciousness and not of anything independent of human consciousness [13]. As described by Mapp [14], phenomenology aspires to construct insight into “lived experience” from the perception of those individuals involved in a particular experience or “phenomenon” [15].

### 2.4. Research Questions

RQ1-Does knowledge and understanding of a family history of diabetes promote positive health behavior changes in African Americans?

RQ2-For individuals who have not made any changes towards prevention, what are the barriers that have prevented change in their lifestyles?

### 2.5. Nature of the Study

A qualitative approach with an emphasis on the HBM and phenomenology was the research design for this study. A qualitative study design enables the researcher to gather data and information from participants by allowing them to express their thoughts and concerns through focus groups and interviews (Patton 2002). This study was centered on recruiting African Americans who self-report a known family history of diabetes but themselves are not diagnosed with the disease. The participants composed a convenience sample of interested African American men and women over the age of 18. Participants were recruited from an inclusion questionnaire that was given to various church congregations in Maryland after receiving permission from the church's pastor allowing dialogue and recruitment from these various churches. Data was collected from face-to-face interviews, transcribed, and analyzed through NVivo.

### 2.6. Participant Selection Logic

African Americans are affected by T2DM more than any other ethnic group [5]. Therefore, individuals who self-identified as African American no matter their place of origin were used in this study. The participants for this research came from the African American population from different churches in the state of Maryland. The counties of interest for the study included Prince George's, Howard, and Montgomery County. Pastors of these churches were approached to discuss the research. The premise of the meetings was to present the information about the research and its importance. Permission was obtained to distribute questionnaires to the churches for their congregations. The pastor in an African American community is held in high regard and is highly respected; hence, building a research partnership with pastors of churches is essential.

### 2.7. Procedures for Recruitment, Participation, and Data Collection

Sampling in qualitative research has a distinct purpose. The sampling strategies and sizes are meant to mirror the diversity of the population being studied and not be a statistical representation [16]. Therefore, purposive sampling method was used. Sampling sizes are usually dictated based upon methods, research questions, and the type of data that is being collected. Most importantly, the sampling size should be significant enough to gather content that is rooted more in depth than in breadth. This often may produce smaller sampling sizes [16]. Similar studies with qualitative methodology and the HBM used smaller samples sizes. Based on the methodology and the type of data collection utilized, sampling was concluded after the first 20 eligible participants.

An inclusion questionnaire was used during the recruitment process to determine eligibility for the study. The main purpose of the inclusion questionnaire was to help discern individuals who self-identified as African Americans and had a family history of T2DM but they themselves had not been diagnosed with the disease. An informed consent was provided to participants prior to any data collection and interviews. The interviews took place in a mutually agreed upon location that was convenient for both parties. Participants were made aware that the interview would be recorded for later transcription and data collection. Video and audio technology from FaceTime or Skype via the internet were utilized as last resorts to conduct the face-to-face interviews when a location to meet in person was not available. Only the audio portion was recorded from a video conference for later transcription and data collection. The collection of data, including the transcriptions and review of the interviews, took place over a period of 6 to 8 weeks. The analysis of the data from the interviews was completed with the use of NVivo Version 12 for Mac computers.

### 2.8. Demographics

The study consisted of six men and 14 women. All the participants self-identified as African American; most reported a birthplace within the United States. Only five participants reported having a birthplace outside of the United States. Although these five participants' birthplaces were located outside of the United States, their time of legal residence within the United States was at least 5 years or more. Participants' ages ranged from 25 to 60 years old. All of the participants lived in either Prince George's, Howard, or Montgomery County in Maryland. Participants' highest level of education completed ranged from “some college” to “graduate degree” (see Table 1).

### 2.9. Data Collection

Data collection began in December 2017 after receiving Institutional Review Board approval (approval number 11-14-17-0165848). Emails were sent to local churches asking to schedule time to speak with the pastor about the study and obtain their permission to recruit participants in their congregation. Four of the seven emails were answered. After speaking in detail with the pastor or the health ministry leader of the church, permission was granted from three churches to make a presentation and distribute inclusion surveys. Participants were required to reside the counties of Prince George's, Howard, or Montgomery. Names of the participants were left out of the recordings and on transcriptions.

### 2.10. Data Analysis

Each response from the interviews was analyzed for common themes. The open-ended questions allowed for elaborate responses. The questions for the interviews were designed to address the following areas of concern for this research study: (1) knowledge of diabetes and prevention, (2) the participant's family history and events around diabetes, and (3) the participant's health behavior. “Knowledge,” “Family History,” and “Personal Health Behavior” were the first three themes created. Another theme that was created was “Barriers.” This theme was created to analyze the responses given to the overall and personal barriers hindering change in health behaviors. Other themes emerged after reading over the transcriptions and discovering common responses within the original themes. Graphs, tables, and figures assisted in the breakdown of the data. Notes were also taken on participant facial expressions and body positioning details.

## 3. Results

### 3.1. Knowledge of Type 2 Diabetes Mellitus

To understand the impact knowledge of a family history of T2DM has on positive health behavior change, it was important to first determine if individuals knew the facts surrounding the disease. Three questions from the interview gauged the overall knowledge of T2DM by assessing its meaning, the signs and symptoms of the disease, and risk factors associated with the disease. Participants had an array of definitions for diabetes, with all of the responses making a connection with T2DM being a disease of the pancreas and the body's inability to process carbohydrates. The most commonly used words or phrases in the definitions included “insulin” or “sugar” in conjunction with the “body's inability to process sugar.” Examples of the responses included “diabetes is a disease that affects the pancreas, or your body's ability to produce or secrete insulin” (Participant 201701) and “the failure of the pancreas to produce enough insulin to efficiently metabolize sugars and carbohydrates” (Participant 201703).

**Figure 1:**
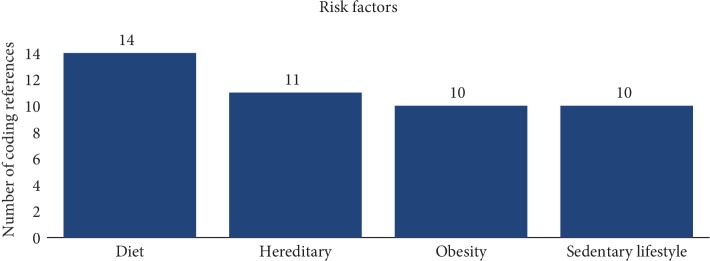
Risk factors associated with T2DM according to study participants.

Individuals were also asked to identify risk factors that were associated with T2DM. The premise behind this question was to help link the awareness that family history is a known risk factor. The most identified risk factors included poor diet, obesity, a sedentary lifestyle, and heredity. Seventy percent of the responses mentioned poor diet as a risk factor while only 55% of the responses recognized there is a hereditary link to developing T2DM (see Figure 1).

**Figure 2:**
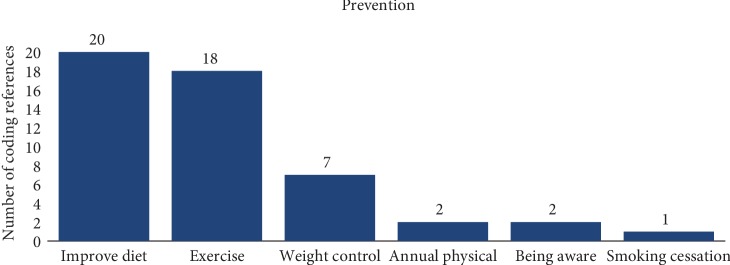
Prevention of T2DM according to the responses of the participants.

Prevention and awareness are important factors when dealing with chronic diseases and public health. The participants were asked to share their knowledge and thoughts surrounding ways individuals could prevent developing T2DM. Overwhelmingly, all of the participants gave a response that either centered around either changing one's diet, increasing physical activity, weight control, or seeing their doctor regularly (see Figure 2). Diet changes included limiting sugar intake or moving from an animal-based to a plant-based diet. Exercising was defined as being involved in cardio-related activities for at least 30 minutes a day for three to five days a week. Several participants' responses reflected on at least three or all of these prevention modalities.

### 3.2. Personal Health Behavior

The HBM suggests that health-related decision-making is determined by (a) perceptions about one's susceptibility to and (b) severity of the illness, (c) perceived benefits of treatment, (d) perceived barriers to seeking care, and (e) cues to action [17, 18]. The questions asked during this study that focused on personal health behavior were able to ascertain one or more of the concepts from the HBM. Participants were first asked to describe their feelings surrounding the fact they had a family member who lived or was living with T2DM. The responses varied from “conscious” to “no feelings at all.” Others had different responses that expressed “sadness” or “no reaction.”

**Figure 3:**
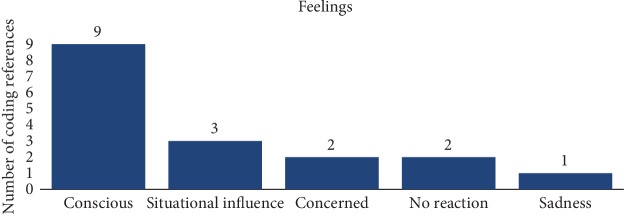
Participants' expressed feeling towards family history of T2DM.

Interestingly, three participants communicated how they felt with describing situational events which occurred in their life with a family member or themselves. Participant 201701 expressed “…there was no concern until their father had a heart attack.” Participant 201703 stated the following: “…then I went to the doctor and they told me that my sugar level was high and that if I didn't do something that I was probably going to be diabetic; so that made me feel like OK, I need to get things in check.” When Participant 201702 saw their family member having to take insulin injections, this was the turning point for them to say “…that was something that I said I do not ever want to have to do…” (see Figure 3).

Personal health behavior also examined the participant's actions as it pertained to the prevention of T2DM. Participants were asked to give details of their own health-related behavior in reference to their own family history of T2DM. Only one participant stated that their knowledge of the disease and their family contributed little to their overall health behavior: “It has had very little influence. I'm sitting here, and I'm embarrassed to say that; it has had very little influence beyond the fact of me being aware.” (Participant 201701). The remaining individuals of the study expressed actions and behavior patterns such as “I purposely watch a lot of what I eat, as far as fat content and sugar content.” (Participant 201708); “I try to exercise regularly and limit my sugar intake, and then I go to get my regular checkups like yearly that include blood work.” (Participant 201711); and “I have significantly reduced my sugar intake; I think the weight gain and knowing my grandfather's history made me a little nervous because I love sugar. I think that's probably the biggest thing I have modified--my diet overall, but sugar has been the thing I have had to pay more attention to” (Participant 201712). Participants were very interested in the study and how they could better their status. Some individuals saw this study as an opportunity to start their change in health for better. Others even saw this study as a wake-up call to how much really is not known about the T2DM.

### 3.3. Barriers

The second research question is an intricate part of this study and to the HBM design. The question relates to the participant's self-awareness of negative behavior or the lack of positive behavior. The interview questions linked to the second research question asked participants to explain in their own words “barriers to change in health” from an overall point-of-view. The participants were then asked to identify any barriers within their personal lives they felt were averting them in making positive health behavior changes or altering negative health behaviors. When asked to define “barriers to change in health,” all of the participants described the phrase as something that hinders change from happening. Participants gave a variety of reasons for barriers within their life. Some stated “self” was the barrier in their own life. One participant eloquently stated, “The biggest barrier or obstacle, I find personally, in my productivity is me—I am my biggest obstacle” (Participant 201701). Participant 201702 expressed the same by saying “My own laziness--the barrier is me just being lazy. I can definitely do better….”

Others found their professional work life, school, or family responsibilities deprived them of time they needed to incorporate positive changes in their schedules. Participant 201708 shared the following: “My barrier right now is time. Right now, I have an hour commute in the morning, and then I work 9-10 hours a day. Then I have another hour commute home. Just enough time to take my daughter to swimming class or spending a couple of hours with her making dinner and then going to bed. I think my personal schedule is the biggest barrier to anything.”

Other participants wanted to invest in their health but realized it would take time, effort, and commitment to maintain things many of the participants did not know how to include in their compressed schedule—“I think free time is a lot of it. …it can take anywhere from an hour to two hours one way to get to work. I would definitely go to the gym if I had more time. If I go to the gym, I have to sacrifice something else” (Participant 201710).

## 4. Discussion

Awareness of a disease's risk factors and preventive measures is paramount for positive health behavior and incorporating lifestyle changes. However, how persons see themselves as being susceptible to developing a disease and how they use the information are the cornerstones of the HBM. This study examined how a family history of T2DM influenced individuals in the African American community in their actions towards positive health behavior change. The study also examined the participant's ideas regarding general and personal barriers to such changes. Whether or not individuals were involved in preventive activities, the participants discussed how barriers played a role in health behavior changes in the general public and for themselves. Each response provided by the participants alluded to how diabetes was the body's inability to process sugar effectively. Many of the participants were able to identify the pancreas was involved in the process of T2DM as the dysfunctional organ. Every individual in the study was also able to give appropriate signs and symptoms of how someone may present with T2DM, which included having extreme thirst, being obese, consuming a diet rich of foods high in fats, sugar, and salt, or having frequent urination. While participants were able to recognize many risk factors associated with the disease, only 11 of 20 of the responses (55%) recognized a family history as being a risk factor for developing T2DM. Much of the responses acknowledged modifiable risk factors such as obesity and a sedentary lifestyle. The latter correlates with the overwhelming answers given from participants with respect to prevention of the disease. Participants' preventive activities focused more on managing weight with a healthy diet and engaging in cardio-related exercises. These findings are in a horizontal alignment with the current research, which shows healthcare providers stress the importance of awareness through modifiable risk factors, whereas family history is rarely emphasized enough during awareness campaigns on the prevention of diabetes [19].

The second theme of the study was centered around personal health behavior. As mentioned before, participants were asked about prevention activities earlier in the study. This question was followed up with a discussion about the participant's health beliefs and behaviors. According to this study, there was broad acknowledgment that more needed to be done by each participant as it pertained to preventive efforts. The proposed activities involved eating a healthier diet with more fruits and vegetables, doing more cardio-related exercises for at least 30 minutes a day, and seeing a healthcare provider annually for a physical and routine checkup. Interestingly, only one participant did not actively engage in any type of the preventive actions mentioned above, although that participant was aware of the potential complication of T2DM. Furthermore, only three participants actively sought out preventive care with annual health visits to their primary care physicians; these findings show a disconnection to the current literature as it pertains to annual health maintenance. Current recommendations for annual health visits have shown benefits with earlier detection in cancers and preventable diseases, along with proper management of chronic diseases [20].

### 4.1. Limitations

There are several limitations to this study. The first limitation of the study was the sample size. Although there is a larger than recommended sample size for a qualitative study, this size does not fairly represent a larger community of African Americans. A second limitation to address in this study was the lack of male participants in the research. Considering African Americans are the least represented race and ethnicity in research, this trend has been noted in other research areas for African American males. The third limitation with this study was that it only asked the experiences of African Americans. Allowing the study to engage anyone with a family history of T2DM may have shown different responses with consideration to culture, preventive healthcare beliefs, and barriers.

## 5. Conclusions

Individuals within the African American community who had a family history of T2DM were the nucleus of this study. The study delved into the communication of health topics within the family and home. Fifty percent of the participants stated health and disease prevention was not a usual topic of discussion in their home growing up. This study could be the catalyst to starting the conversation of disease prevention in the home at an earlier age. Butler and Mead [21] both suggest beginning preventive health messages during the earliest time of developing lifelong habits. The idea of preventive messages within the home at an earlier age feeds into the idea of changing the mind of generations to come. This study provides a sense of generational awareness of T2DM and the critical impact family history has on disease prevention. The study brings to the forefront the need to address health topics not only in the doctor's office but also more in the home and communities. Health communication strengthens awareness, which in hope encourages action for positive health behavior change at an earlier age.

Awareness, just like communication, is twofold. Awareness campaigns for the community are developed with strategic plans in place with all stakeholders in mind. Policymakers, being a significant contributor to the class of stakeholders, are intricate to the implementation of positive social change. Many African Americans communities are stricken with poverty and less than favorable access to healthcare. Policymakers should be made aware of the challenges such communities face. To fully comprehend the barriers affecting many communities, the difficult conversations between those affected and those who can help with change need to happen more often face-to-face. Just as this study was able to highlight barriers for participants, town hall meetings should occur to have the voices of the communities heard by the policymakers. Change can only take place if people are willing to accept the fact barriers exist and are disproportionate to various communities. Social determinates play a major role in access to healthcare, knowledge and understanding of disease awareness and prevention, and an individual's overall health outcome to chronic disease. More specifically, in communities where socioeconomic disadvantages are the significant barriers, it is proposed that policymakers and public health officials should gather in communities to hear firsthand how healthier eating options are not as available or affordable in some communities. The options to eat healthier are at times far more expensive to families who may be struggling financially versus others who are able to afford a desired meal plan that is organic, plant-based, or vegan [22]. Awareness to every component of this multifaceted problem is the key to proper change.

Social change also pertains to building and maintaining meaningful relationships between the health providers and the communities they serve. This study sheds light on how one can strengthen the healthcare provider and patient relationship, especially among the African American community. African Americans have a deep history of mistrust from health providers dating back to the Tuskegee Experiments [23]. This mistrust of care has planted seeds of doubt for many generations. Specifically, healthcare providers can use the interview questions as a guide to engage their patients to be open about their knowledge of a disease, their family history, their own healthcare practices or beliefs, and personal barriers within their lives which inhibit positive health behavior changes. Furthermore, providers should allow their patients to be more involved in their healthcare decision-making and tailor an action plan to their current lifestyle and recognizable barriers.

According to the CDC, there are more than 29 million people living in America who are diagnosed with T2DM and nearly 60 million individuals who are prediabetic [2]. This puts a financial burden on the healthcare system which paid $245 billion only for T2DM and complications associated with the disease in 2012 [24]. The predicted increase in prevalence, especially among the African American community, has made diabetes a public health concern. The increase has set off a chain reaction of care planning. The main goal for T2DM in the public health realm is geared towards awareness. Awareness focuses on bringing attention to modifiable risk factors. The hope is that awareness will promote preventive actions with lifestyle modification for obesity and a sedentary lifestyle. These modifications include increasing physical activity and eating a healthy diet. Furthermore, awareness campaigns should also shed light on nonmodifiable risk factors, such as a family history of T2DM. Preventative actions can still be put into place while encouraging individuals to visit their healthcare providers routinely to have blood work done which could detect the disease.

This study, with the limitations mentioned, proved to show an individual's family history of T2DM was a strong influence in positive health behavior change for the participants involved. Continuing to focus on awareness and prevention, this study subscribes to the idea of making family history, a nonmodifiable risk factor, just as important as modifiable risk factors. Family history, although it is not a modifiable risk factor, should be added to the awareness campaign. Having providers and patients understand the overall dangers of T2DM with an added focus on family history can be beneficial to all involved. The topic of family history opens the pipe lines of discussion not only for T2DM but also for other healthcare concerns. The provider and patient relationship becomes stronger than ever and flourishes.

Barriers became a topic of discussion as well. Individuals within this study were able to realize the true definition of a barrier and how barriers impacted their desires to do better. Barriers for these participants mainly dealt with time. Fortunately, access to healthy food or a gym membership was not a part of the barriers to change in health for this study. The main goal to overcoming barriers was to set realistic expectations and start small. Setting realistic action plans and expectations aids in an overall better outcome. Furthermore, individuals had a chance to be honest about these goals while taking into considerations their acknowledged barriers. Not all barriers can be modified. However, recognizing that barriers exist and having the difficult conversations between communities and all stakeholders are the beginning many are looking for to start a new healthy lifestyle.

## Figures and Tables

**Table 1:** Frequencies and percentages for participant demographics.

Demographic characteristics	Frequency *N* = 20	Percentage
Gender		
Male	6	30
Female	14	70
Origin of birthplace		
USA	15	75
Other	5	25
Highest level of education completed		
Some college	3	15
Bachelor's degree	8	40
Master's degree	6	30
Ph.D./PharmD/DPT	3	15

## Data Availability

The data used to support the findings of this study are available from the corresponding author upon request.
